# Promoting Physical Activity during School Closures Imposed by the First Wave of the COVID-19 Pandemic: Physical Education Teachers’ Behaviors in France, Italy and Turkey

**DOI:** 10.3390/ijerph17249431

**Published:** 2020-12-16

**Authors:** Erica Gobbi, Silvio Maltagliati, Philippe Sarrazin, Selenia di Fronso, Alessandra Colangelo, Boris Cheval, Géraldine Escriva-Boulley, Damien Tessier, Giyasettin Demirhan, Gokce Erturan, Yilmaz Yüksel, Athanasios Papaioannou, Maurizio Bertollo, Attilio Carraro

**Affiliations:** 1 Department of Biomolecular Sciences, University of Urbino Carlo Bo, 61029 Urbino, Italy; erica.gobbi@uniurb.it; 2 School of Human Movement & Sport Sciences, Université Grenoble Alpes, SENS, F-38000 Grenoble, France; silvio.maltagliati@univ-grenoble-alpes.fr (S.M.); philippe.sarrazin@univ-grenoble-alpes.fr (P.S.); geraldine.escriva-boulley@univ-grenoble-alpes.fr (G.E.-B.); damien.tessier@univ-grenoble-alpes.fr (D.T.); 3 Department of Medicine and Aging Sciences, University G. d’Annunzio of Chieti and Pescara, 66100 Chieti, Italy; s.difronso@unich.it; 4 Department of Philosophy, Sociology, Education and Applied Psychology, University of Padova, 35137 Padova, Italy; alessandra.colangelo@phd.unipd.it; 5 Swiss Center for Affective Sciences, Laboratory for the Study of Emotion Elicitation and Expression (E3Lab), Department of Psychology, University of Geneva, 1202 Geneva, Switzerland; Boris.Cheval@unige.ch; 6 Department of Physical Education and Sports Teaching, Faculty of Sport Sciences, Hacettepe University, 06800 Ankara, Turkey; demirhan@hacettepe.edu.tr (G.D.); yyuksel@karabuk.edu.tr (Y.Y.); 7 Department of Physical Education and Sports Teaching, Faculty of Sport Sciences, Pamukkale University, 20020 Denizli, Turkey; agerturan@pau.edu.tr; 8 Hasan Dogan School of Physical Education and Sport, Karabük University, 78050 Karabük, Turkey; 9 Department of Physical Education and Sport Sciences, University of Thessaly, 42100 Trikala, Greece; sakispap@uth.gr; 10 Faculty of Education, Free University of Bozen-Bolzano, 39042 Brixen, Italy

**Keywords:** COVID-19, lockdown, online teaching, physical education, physical activity, teachers, secondary school

## Abstract

The COVID-19 pandemic has drastically reduced physical activity (PA) behaviors of many people. Physical education (PE) is considered one of the privileged instruments to promote youths’ PA. We aimed to investigate the effects of lockdown on PE teachers’ behaviors promoting their students’ out-of-school PA and differences between three European countries. A sample of 1146 PE teachers (59.5% females) from France, Italy, and Turkey answered an online questionnaire about guiding students to engage in out-of-school PA, helping them to set PA goals, encouraging in self-monitoring PA, the pedagogical formats of these behaviors and feedback asked to students. RM-MANCOVAs were performed with a two-time (before and during the lockdown), three country (France, Italy, Turkey), two gender factorial design, using teaching years and perceived health as covariates. A significant multivariate main effect time × country × gender (*p* < 0.001) was reported for the behaviors promoting students’ PA, with French and Italian teachers increasing some behaviors, while Turkish teachers showing opposite trends. Significant multivariate main effects time × country were found for formats supporting the behaviors (*p* < 0.001) and for asked feedback formats (*p* < 0.001). The massive contextual change imposed by lockdown caused different reactions in teachers from the three countries. Findings are informative for PA promotion and PE teachers’ education.

## 1. Introduction

**Figure 1:**
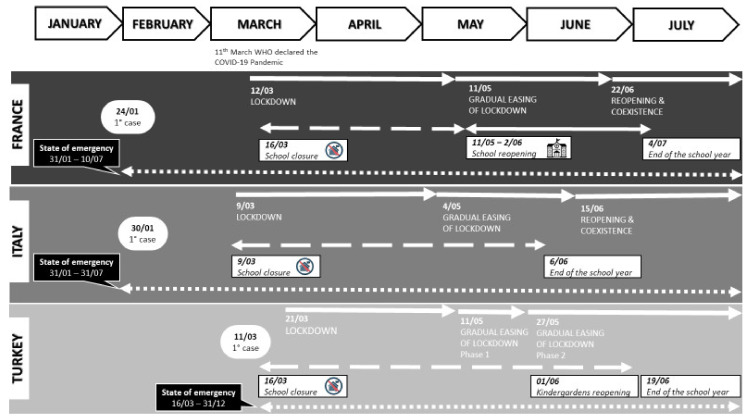
Lockdown phases and school closures in France, Italy, and Turkey.

On 11 March 2020, the World Health Organization (WHO) declared the Coronavirus (COVID-19) outbreak a global pandemic. In many countries, to limit the viral spread, people have been asked to radically change their lifestyle: to isolate themselves or live confined at home, to restrict exercise, to work from home, and to cope with the temporary closure of schools, universities, and non-essential businesses. In France, Italy, and Turkey, the countries in which the present study was conducted, different lockdown actions have been implemented. French and Italian populations of all ages were confined at home for about eight weeks, from March to May 2020. In Turkey, people over 65 years had to stay at home in March and April, while the same restriction was imposed to children and youths under 20 years only in April. This restriction was extended to the entire population on some specific days (e.g., weekends and religious holidays) in April and May (Figure 1).

This prolonged lockdown has been detrimental for individuals’ physical and mental health [1,2,3]. In particular, the imposed restrictions have negatively affected several life domains among young people, although the direct health impact of COVID-19 seems lower in this population than among older adults. Youths reported reduced health-related quality of life [4], negative psychological and emotional outcomes [5,6,7], behavioral difficulties such as sleeping problems [8], poor nutritional regimen [9], increased sedentariness and screen-time [10,11].

To counteract such detrimental consequences, the WHO encouraged people of all ages to “be active and stay healthy at home” [12]. It is well known that regular physical activity (PA—i.e., at least 150 min/week for adults and 1 h/day for children and adolescents [13]) enables people to limit numerous risk factors for physical and mental diseases [14,15,16] and promotes individual’s well-being [17]. Unfortunately, strong evidence reveals that the majority of children and adolescents in many countries do not participate in sufficient PA [18]. Moreover, as observed among adults [19], adolescents’ PA levels have been shown to decline during lockdown, probably because of the closure of schools, sports facilities, and public parks [20]. In this scenario, the promotion of PA participation among young people has represented a public health priority.

Physical education (PE) is considered one of the privileged instruments to promote youths’ PA, both inside school [21,22,23] and out-of-school [24,25,26]. PE has the potential to reach all students since it is a compulsory school subject—at least in Europe—providing them with the acquisition of motor skills and behavior transferable to different life contexts [21,27,28,29]; these goals are highlighted in the national PE curricula of France, Italy, and Turkey. Moreover, unlike other exercise settings, such as sport clubs and associations, PE is taught by qualified teachers who are assumed to create contexts fostering positive values, securing students’ engagement and promoting positive development [30,31]. During the first wave of the COVID-19 pandemic, more than ever, PE represented a promising mean to promote out-of-school PA. In the meantime, because of school closures, teachers faced an unprecedent challenge: to promote PA while lacking direct contact with their students. The role of PE in promoting PA during the lockdown was also emphasized to a different extent in the countries involved in the present study. In France, this role was nationally acknowledged by national authorities, through the publications of guidelines. In Italy, support and recommendation came from school principals and boards, without any specific suggestions or instructions from the Ministry of Education. In Turkey, online general education was provided via a free-access TV channel named EBA. However, PE classes were not part of it: PE teachers were not obliged to teach their classrooms during lockdown, although some of them did. Regardless of the country, this situation forced PE teachers to cope with crucial educational issues: “What pedagogical behaviors could be adopted in order to promote students’ out-of-school PA during school closures? And in what pedagogical format?” Due to their influence on self-regulatory skills in PA, three teachers’ behaviors can be considered of key importance to promote students’ out-of-school PA [32,33]: (a) guiding students to engage in out-of-school PA; (b) helping them to set personal PA goals; and (c) encouraging to self-monitor PA [29,34,35]. Another important feature of students’ PA promotion lies in teachers’ propensity to ask students feedbacks about their PA level. Nevertheless, the implementation of online teaching, subsequent to school closures, brought a significant disruption in PE teachers’ habitual teaching behaviors, which were usually based upon face-to-face, body-centered teaching modalities. Therefore, it appears important to understand the evolution of the pedagogical formats on which such behaviors were based upon.

Consequently, the purpose of the present study was twofold. First, it aimed to examine, from before to during the COVID-19 lockdown, the changes of French, Italian, and Turkish PE teachers’ behaviors promoting their students’ out-of-school PA, alongside with the changes of pedagogical formats in relation to these behaviors. Second, because educational settings differed between the three countries involved, we explored whether the possible changes in teachers’ behaviors due to lockdown were depended on the considered country, or on teachers’ gender [36]. Moreover, since years of teaching [37] and perceived physical and mental health status [38,39] could have influenced the outcomes, they were considered as covariates in the analyses. To the best of our knowledge, this is the first study focusing on the changes of PE teachers’ behaviors promoting students’ out-of-school PA during the school closures imposed by the COVID-19 pandemic. Consequently, the study is exploratory in its nature and no a priori hypothesis based on previous literature could be made.

## 2. Materials and Methods

### 2.1. Study Design

Mixed factorial design between-within subjects with a retrospective assessment of variables for the before-lockdown period.

### 2.2. Procedure

In France, Italy, and Turkey, PE teachers’ answers were obtained using an anonymous questionnaire. Data were collected from the end of April to the middle of May 2020, corresponding to the school closure period in the three involved countries. The questionnaire was administered through online survey platforms (i.e., Google Forms and LimeSurvey), and accessed once by participants using a designated link. The latter was disseminated through PE teachers’ social networks and using the snowball sampling technique.

The study was developed in accordance with the principles of the Declaration of Helsinki for the protection of human rights. Answering the questionnaire, all the participants provided an informed consent expressing their voluntary participation and agreed with the analysis and use of the resulting data. Participants could interrupt or quit the survey at any point without explaining the reasons for doing so. The study obtained the approval of the Turkish Ministry of Health Scientific Research Platform (approval no. 2020-05-30T09_25_01) and Hacettepe University Ethics Committee (approval no. 35853172-050.06).

### 2.3. Participants

Participants were 1146 secondary school PE teachers (59.5% females) from France (*n* = 434), Italy (*n* = 497), and Turkey (*n* = 215). Sociodemographic data and perceived physical and mental health are summarized in Table 1.

### 2.4. Measures

The questionnaire was developed in English, then translated and administered in each country language. It took approximately 20 min to be completed and is available in Appendix S1. To reduce comparison bias, in the first part of the questionnaire participants were requested to provide information on the variables of interest before the lockdown (e.g., “This part of the questionnaire focuses on your behaviors before the lockdown period”). In the second part of the questionnaire, participants were asked to provide information on the variables of interest during the lockdown (e.g., “This part of the questionnaire focuses on your behaviors during the lockdown period”). The covered areas comprised (a) behaviors aiming at promoting students’ out-of-school PA; (b) pedagogical format supporting these behaviors; (c) frequency and pedagogical format of asked feedback about students’ out-of-school PA levels. Finally, sociodemographic data and perceived health were collected as possible confounding variables. Except for sociodemographic data and perceived health, all variables were assessed using five-point Likert scales ranging from 1 (never) to 5 (always).

#### 2.4.1. Sociodemographic Data and Perceived Health

Sociodemographic data included gender, age, and years of PE teaching (Table 1). Perceived health was assessed using two items from the Patient-Reported Outcomes Measurement Information System (PROMIS) [40]. One item assessed physical health and one item measured mental health. Both items referring to the last seven days and scored on a four-point Likert scale ranging from 1 (poor) to 4 (excellent).

#### 2.4.2. Behaviors Promoting Students’ Out-of-School PA

This part of the questionnaire captured self-reported teachers’ behaviors promoting students’ out-of-school PA, before and during lockdown. In the absence of validated tools, three items were created in order to collect how frequently teachers were supporting their students in three fundamental dimensions of self-regulated out-of-school PA. Specifically, teachers were asked to indicate how often they: (a) guide/d students to engage in out-of-school PA; (b) help/ed them to set personal PA goals; and (c) encourage/d their students to self-monitor PA.

#### 2.4.3. Pedagogical Formats of Behaviors Promoting Students’ Out-of-School PA

A second series of questions assessed how often PE teachers promoted students’ out-of-school PA using several pedagogical formats, before and during lockdown. Teachers were invited to answer: “If you propose/d pedagogical contents aiming to promote out-of-school PA among your students, in what format and how often do/did you propose them?” After this stem, four types of pedagogical formats were proposed: text documents (e.g., notebooks); slideshows (e.g., posters); videos (i.e., video tutorials with exercises to do at home); and live streaming lessons (e.g., online PE classes delivered in live streaming). These formats were selected based upon their possible usefulness in the context of distance teaching.

#### 2.4.4. Frequency and Pedagogical Formats of Asked Feedback about Students’ Out-of-School PA

This part of the questionnaire measured self-reported teachers’ frequency in asking students’ feedback about their out-of-school PA. A one-item question was proposed: “If you propose/d pedagogical contents aiming to promote outside-school PA among your students, how often do/did you ask them to make some feedback about what they have/had actually done?”

A second series of questions also assessed how often teachers used several pedagogical formats to ask their students some feedback about their out-of-school PA. Three types of pedagogical feedback formats were proposed: text documents (e.g., notebooks), smartApps (e.g., summary records from smartphone apps), pictures and/or videos.

### 2.5. Statistical Analysis

Data were initially screened for outliers [41], that were no detected. Examination of histograms, skewness, and kurtosis of the scored variables showed that there was no substantial deviation from normal distributions. While controlling for sociodemographic variables (i.e., years of PE teaching, and perceived physical and mental health), a series of mixed between-within Repeated Measures Multivariate Analysis of Covariance (RM-MANCOVA) was performed, with a two-time (before vs. during the lockdown), three-country (France vs. Italy vs. Turkey), two-gender (male vs. female) factorial design. These analyses were computed for the dimension of teachers’ behaviors promoting students’ out-of-school PA, for the pedagogical formats of such behaviors, and for the asked feedback about students’ out-of-school PA. Bonferroni correction test was used for post-hoc pairwise comparisons. Effect sizes were calculated using partial eta square (η_p_^2^) [42] in the analyses of variance, with 0.01, 0.06, and 0.14 considered small, medium, and large effects, respectively [43]. The significance level was set at *p* < 0.05. Statistical analyses were performed using the STATISTICA 12 software (StatSoft, Inc., Tulsa, OK, USA).

## 3. Results

### 3.1. Teachers’ Behaviors Promoting Students’ Out-of-School PA

RM-MANCOVA revealed that years of teaching PE (*p* = 0.015), perceived physical (*p* = 0.002) and mental health (*p* = 0.003) significantly adjusted values of teachers’ behaviors in promoting students’ out-of-school PA (Table S1).

When controlling for these covariates, significant multivariate main effects Time × Country (Wilk’s λ = 0.737, F (6, 2270) = 62.2242, *p* < 0.001, ηp^2^ = 0.141, Power = 1) and Time × Country × Gender were found in teachers’ behaviors (Wilk’s λ = 0.982, F (6, 2270) = 3.360, *p* = 0.003, ηp^2^ = 0.009, Power = 0.942).

**Figure 2:**
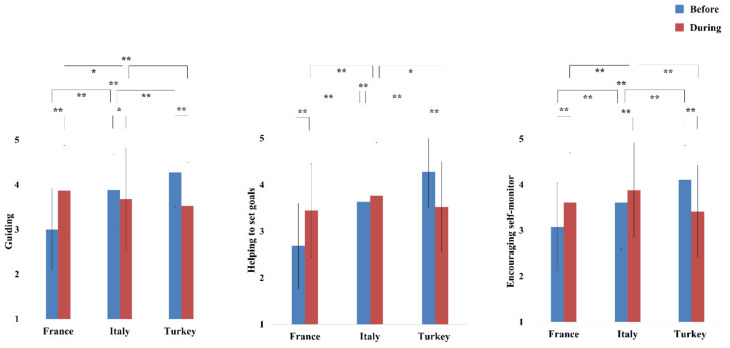
Time × country interactions in the three analyzed teachers’ behaviors. Note. Means and standard deviations are represented. *: *p* < 0.05; **: *p* < 0.01.

Follow-up univariate ANOVAs showed significant interactions Time × Country in all the three analyzed teachers’ behaviors (Figure 2): guiding students to engage in out-of-school PA (F (2, 1137) = 147.691, *p* < 0.001, ηp^2^ = 0.206, Power = 1), helping students in setting their PA goals (F (2, 1137) = 113.564, *p* < 0.001, ηp^2^ = 0.167, Power = 1), and encouraging students in self-monitoring PA (F (2, 1137) = 74.465, *p* < 0.001, ηp^2^ = 0.116, Power = 1). Post-hoc pairwise comparisons using Bonferroni correction are shown in Table 2.

From before to during lockdown, French teachers significantly increased each of the three behaviors (ps < 0.001). Italian teachers increased the encouragement of self-monitoring (*p* < 0.001), reduced guidance (*p* < 0.01), while no significant difference was observed for the promotion of goal-setting. Turkish teachers significantly reduced the frequency of all the three behaviors (*p* < 0.001).

Before lockdown, differences between countries were found, with French teachers showing lower frequency in each of the three behaviors, in comparison to Italian and Turkish teachers (*p* < 0.001). Turkish teachers reported a higher frequency in all the three behaviors than Italian and French teachers (*p* < 0.001). During lockdown, results showed that French teachers reported a higher frequency of behaviors guiding students to engage in PA (*p* < 0.01) compared to those of teachers from the two other countries, which did not differ from each other. Italian teachers manifested a higher frequency in behaviors aiming to goal-setting and encouraging self-monitoring than French (ps < 0.001) and Turkish teachers (*p* < 0.05 and *p* < 0.001 respectively), which did not differ one from each other.

Follow-up univariate ANOVAs showed significant interactions time × country × gender in guiding students out-of-school PA (F (2, 1137) = 8.558, *p* < 0.001, ηp^2^ = 0.015, Power = 0.967) and helping students in setting their PA goals (F (2, 1137) = 4.527, *p* = 0.011, ηp^2^ = 0.008, Power = 0.772). No effect was found for encouraging students in self-monitor PA (*p* = 0.354). Post-hoc pairwise comparison results using Bonferroni correction in the interaction effect time × country × gender are reported in Table 2. Post-hoc analyses referred to the time × country × gender interaction highlighted a significant difference by gender among Italian teachers, with women reporting higher frequency in guiding students during lockdown, in comparison to their male counterparts (*p* < 0.05).

### 3.2. Pedagogical Formats of Behaviors Promoting Students’ Out-of-School PA

RM-MANCOVA revealed that perceived physical health (*p* < 0.001) significantly adjusted scores for pedagogical formats, while no significant effects were found for years of teaching PE (*p* = 0.199) and perceived mental health (*p* = 0.067) (Table S2).

When controlling for these covariates, a significant multivariate main effect, time × country was found in pedagogical formats (Wilk’s λ = 0.653, F (8, 2268) = 67.399, *p* < 0.001, ηp^2^ = 0.192, Power = 1), while no significant multivariate main effect time × country × gender was highlighted (*p* = 0.112). Descriptive statistics are reported in Table 3.

Follow-up univariate ANOVAs showed significant interactions time × country in text documents (F (2, 1137) = 119.895, *p* < 0.001, ηp^2^ = 0.174, Power = 1), slideshow (F (2, 1137) = 82.240, *p* < 0.001, ηp^2^ = 0.126, Power = 1), videos (F (2, 1137) = 145.109, *p* < 0.001, ηp^2^ = 0.203, Power = 1), and live streaming lessons (F (2, 1137) = 32.105, *p* < 0.001, ηp^2^ = 0.053, Power = 1). Post-hoc pairwise comparison results using Bonferroni correction are presented in Table 3.

From before to during lockdown, French and Italian teachers increased the use of all the pedagogical formats of behaviors promoting students’ out-of-school PA (*p* < 0.05), with the exception of the use of slideshows for Italian teachers (*p* > 0.999). Turkish teachers increased the use of live streaming lesson (*p* = 0.001) and decreased the use of text documents and slideshows (*p* < 0.001), while no significant difference was observed for the use of video tutorials (*p* = 0.348).

Before lockdown, French teachers reported lower frequency in all the four pedagogical formats in comparison to Italian and Turkish teachers. Turkish teachers reported higher frequency in all the pedagogical formats. During lockdown, French and Italian teachers reported higher frequency in using text documents than Turkish (*p* < 0.001). Italian teachers reported higher frequency in using slideshows (*p* < 0.001) and videos than French and Turkish teachers (*p* < 0.001), which did not differ from each other (*p* > 0.999). Italian and Turkish teachers (which did not differ from each other, *p* > 0.999) reported higher frequency of use of live streaming lessons than French teachers (*p* < 0.001).

### 3.3. Frequency and Formats of Asked Feedback about Students’ Out-of-School PA

RM-MANCOVA revealed that only perceived physical health (*p* = 0.008) significantly adjusted values of asked feedback about students’ out-of-school PA. No significant effect of years of teaching PE (*p* = 0.312) and perceived mental health (*p* = 0.093) were found (Table S3).

When controlling for these covariates, a significant multivariate main effect time × country was found in frequency of asked feedback (Wilk’s λ = 0.769, F (8, 2268) = 39.889, *p* < 0.001, ηp^2^ = 0.123, Power = 1). No significant multivariate main effect time × country × gender was reported (*p* = 0.081). Descriptive statistics are reported in Table 4.

Follow-up univariate ANOVAs showed significant interactions Country × Time in feedback frequency (F (2, 1137) = 90.342, *p* < 0.001, ηp^2^ = 0.137, Power = 1), feedback with text documents (F (2, 1137) = 92.352, *p* < 0.001, ηp^2^ = 0.140, Power = 1), feedback by smartApps (F (2, 1137) = 34.088, *p* < 0.001, ηp^2^ = 0.057, Power = 1), and feedback with videos/pictures (F (2, 1137) = 63.752, *p* < 0.001, ηp^2^ = 0.101, Power = 1).

Post-hoc pairwise comparison results using Bonferroni correction showed that, from before to during lockdown, French and Italian teachers increased the frequency of asked feedback about students’ out-of-school PA (*p* < 0.001), while the Turkish decreased it (*p* < 0.001). Also, French and Italian teachers reported an increase in the use of all the different feedback formats (ps. < 0.001). Turkish teachers decreased the use of text documents (*p* = 0.01), while no significant differences were observed for smartApps (*p* > 0.999) and pictures/videos (*p* = 0.277).

Before lockdown, French teachers reported lower frequency of asked feedback (*p* < 0.001) and the use of the three formats (*p* < 0.001) in comparison to Italian and Turkish teachers (*p* < 0.001). Turkish teachers reported higher frequency of asked feedback (*p* < 0.001) and the use of the three formats (*p* < 0.001) than both Italian and French teachers. During lockdown, Italian teachers reported higher frequency of asked feedback (*p* < 0.001) in comparison to French and Turkish teachers, which did not differ from each other (*p* > 0.999). Regarding the format of feedback, two different patterns were reported. First, French and Italian teachers, which did not differ from each other, (*p* > 0.999) reported higher use of text documents than Turkish teachers (*p* = 0.006 for French and *p* < 0.001 for Italian teachers). Second, Italian and Turkish teachers, which did not differ from each other (*p* = 0.319 for SmartApps and *p* = 0.207 for pictures/videos) reported higher use of smartApps and pictures/videos than French teachers (ps. < 0.001).

## 4. Discussion

Throughout school closures imposed in many European countries by the first wave of the pandemic, PE teachers faced the big challenge to replace traditional in-presence teaching with online teaching, thereby radically transforming discipline contents, methodologies, practices, and communication strategies. The present study examined the changes in three PE teachers’ behaviors promoting students’ out-of-school PA (i.e., guiding students to engage in out-of-school PA, helping to set realistic goals, and encouraging self-monitoring, asking students for feedback) and pedagogical formats supporting these behaviors, in France, Italy, and Turkey. Main findings showed significant differences in the changes of these variables across the three countries. From before to during the school closures period, French teachers increased the frequency of all the three analyzed behaviors. Italian teachers reported an increase in helping students to set PA goals, a decrease in guiding students’ out-of-school PA and no significant difference in encouraging self-monitoring PA. Turkish teachers reported a decrease in all the three behaviors. These differences could be interpreted considering the national guidelines related to PE implemented in each of the three countries during lockdown. In France, PE teachers were asked to contribute to maintain a pedagogical continuity and encouraged to use a broad range of digital work environments. Moreover, educational authorities supported the key role of PE in fostering an active lifestyle to counteract the adverse consequences associated with lockdown [44]. Similarly, in Italy, online PE replaced traditional face-to-face teaching [45], but teachers did not receive specific instructions or suggestions from the Ministry of Education about the role of PE in this context. Italian PE teachers taught online classes following recommendations given by school principals and boards, organizing both synchronous and asynchronous classes, using different online teaching platforms and integrating social media. In Turkey, online education was provided via a free-access TV channel named EBA, an online portal that enabled teachers to share educational materials. Although EBA TV covers many disciplines in the curriculum of all school grades, PE classes were not included. In Turkish public schools, PE teachers were not obliged to teach their classrooms during lockdown. Nevertheless, some of them tried to stay in contact with students, providing tutorials, online lessons or uploading materials in the online EBA system. This disparity between countries in terms of the educational role and expectations from PE may have contributed to explain the changes in teachers’ behaviors promoting students’ out-of-school PA. The general increase in French teachers’ behaviors may suggest that PE benefited from a broader approach related to health promotion. In an opposite trend, the situation observed in Turkey was probably related to the difficulty to deliver any lesson, even online. In the perspective of the self-determination theory [46], previous findings suggested that policy makers and governments should support teachers through the satisfaction of the three basic needs (i.e., autonomy, competence, and relatedness), in order to improve teachers’ involvement in educational innovations [47]. Although speculative, our findings consistently showed that when teachers were supported by the educational system, or locally by their principals and/or school boards, they tend to adopt more adaptive behaviors (i.e., to engage in the challenge to promote out-of-school PA during the lockdown). Conversely, without clear guidelines and support (as in the case of Turkish and partially of Italian teachers), teachers may feel more reluctant to adapt themselves to an unprecedented situation, leading to a weaker engagement in the promotion of out-of-school PA.

These distinct changes of PE teachers’ behaviors across countries may also be explained by the initial familiarity with the promotion of out-of-school PA. When observing the comparisons by country, before lockdown, French teachers reported significantly lower values in all the behaviors in comparison to Italian and Turkish colleagues. In this perspective, one previous study showed that PE French teachers favored the acquisition of motor skills over the promotion of out-of-school PA [48]. Accordingly, by depriving them from their habitual objectives, it seems plausible that the lockdown period may have created a shift in PE curriculum goals, moving from practical skills acquisition toward the promotion of out-of-school PA. By contrast, it seemed that, before lockdown, Italian and Turkish teachers were more used to promote out-of-school PA among their students. However, the difficulties related to this new teaching situation (e.g., online teaching and restrictions for students in exercising at home) may have impeded the delivery of the traditional curricula, which in Italy and Turkey include specific references to out-of-school PA promotion [49,50,51,52].

Regarding pedagogical formats used by teachers to promote out-of-school PA (i.e., text documents, slideshows, videos, live streaming lessons) and to ask feedback to their students (i.e., text documents, smartApps, pictures, videos), a similar trend was reported in France and Italy from before to during the lockdown. Teachers manifested a significant increase in the frequency of all the formats proposed, except slideshows for Italian teachers. This means that French and Italian PE teachers tried to adapt to the new situation by renovating their pedagogical formats to promote their students’ out-of-school PA. Differently, among Turkish teachers, a significant decrease was found in the use of text documents and slideshows formats to promote out-of-school PA. During lockdown, they reported lower frequency of asked feedback than usual, in general and specifically by using text documents. An opposite tendency was the increase of live streaming lessons (probably caused by a portion of teachers who decided to deliver online teaching), a pedagogical support which was already part of the traditional curriculum [53].

Another result is the gender effect found only among Italian teachers in guiding students out-of-school PA. Specifically, from before to during lockdown, male teachers decreased their engagement in this behavior, while no significant difference was observed among female teachers. We could hypothesize that Italian male teachers perceived online teaching as a greater barrier to promote out-of-school PA than woman did. In support of this assumption, Collie et al. [54] found that relatedness with students—greatly disrupted by online education—was positively associated with the ability to successfully manage common challenges in everyday work only for male teachers. Further research is needed in order to examine whether male and female teachers differ in their adaptation to a massive educational disruption, such as the COVID-19 lockdown.

This study has several strengths. First, it provides, for the first time, an overview on the changes, from before to during lockdown, of PE teachers’ behaviors promoting out-of-school PA. Moreover, it provides an international perspective on the situation, with a large sample composed of teachers from three different countries. However, this study also presents some limitations. First, it did not provide evidence on the effectiveness of teachers’ behaviors in promoting out-of-school PA among students. Second, the use of self-reported questionnaires and the fact that the variables before lockdown were retrospectively assessed could have enhanced the risk of recall bias. Third, this study does not provide information about the motivational variables which could explain PE teachers’ engagement in the challenge to promote out-of-school PA during lockdown. Future research could aim to investigate PE teachers’ behaviors promoting PA among their students in relation to specific personal aspects. Multilevel analysis studies could help understanding mechanisms and variables that explain changes in teachers’ behaviors. Further research is needed to better understand underlying mechanisms explaining the observed changes of teachers’ behaviors.

## 5. Conclusions

The changes of behaviors promoting students’ out of school PA and of the related pedagogical formats reflect that PE teachers reacted differently in the three involved countries to the unprecedented challenge raised by the first wave of the COVID-19 pandemic. French teachers increased all the behaviors to promote out-of-school PA in their students; Italians increased the behavior in helping students to set PA goals but decreased the guiding of out-of-school PA; Turkish teachers decreased all the behaviors. Findings were likely to be related to the policies adopted by national governments and educational bodies. In France, the role of PE in the promotion of PA was emphasized during lockdown, which was not (fully) the case in Italy and Turkey. Although further research is yet needed to better understand underlying mechanisms explaining the observed changes, our findings highlight the importance of national and local authorities’ in supporting the adaptation of teachers’ behaviors. Finally, several associations recently published guidelines to help stakeholders, school principals, and teachers to ‘reconstruct’ and redesign PE during the COVID-19 pandemic [55,56,57]. These recommendations consider PE standards, but also identify resources and creative ideas that could help PE teachers to reach their learning goals, while respecting safety norms and specific settings/conditions. The present findings also inform on the capacities and adaptability of teachers to an unprecedented teaching situation; however, educational associations and boards should consider the opportunity to pay attention on better preparing teachers to promote out-of-school PA, for instance by fostering the use of smartApps designed to deliver contents and receive feedback [58]. It is probably needed to consider the COVID-19 pandemic as an ‘opportunity’ to restore the role of PE in youths’ lives, with regards to the promotion of PA.

## Figures and Tables

**Table 1:** Sociodemographic data and health perception of PE teachers, by country and gender.

Country	Gender	*n* (%)	Age	Years of PE Teaching	Perceived Physical Health	Perceived Mental Health
France	Male	221 (50.9%)	42.9 (11.3)	19.7 (11.3)	2.9 (0.8)	2.8 (0.8)
	Female	213 (49.1%)	40.5 (10.2)	16.6 (9.8)	3.1 (0.8)	2.9 (0.7)
Italy	Male	158 (31.8%)	50.4 (10.1)	21.1 (13.5)	3.0 (0.6)	2.9 (0.7)
	Female	339 (68.2%)	49.8 (10.5)	21.4 (13.1)	2.9 (0.7)	2.7 (0.8)
Turkey	Male	85 (39.5%)	40.0 (8.7)	16.6 (9.9)	3.1 (0.7)	2.9 (0.7)
	Female	130 (60.5%)	41.9 (9.4)	16.6 (10.3)	3.0 (0.7)	2.9 (0.7)

Note: Data are expressed as mean (standard deviation). Perceived physical and mental health were captured on a scale ranging from 1 to 4.

**Table 2:** Descriptive statistics and Bonferroni post-hoc pairwise comparisons of the interactions time × country × gender in the three teachers’ behaviors promoting students’ out-of-school PA.

Country	Gender	Guiding Students		Setting PA Goal		Self-Monitoring PA	
		Before	During	Before	During	Before	During
**France**	**M**	2.99 (0.93) *^,i,t^	3.95 (0.99) *^,i^	2.65 (0.95) *^,i,t^	3.47 (1.10) *	3.08 (0.99) *^,i,t^	3.68 (1.11) *
	**F**	3.01 (0.92) *^,i,t^	3.79 (1.04) *^,t^	2.73 (0.96) *^,i,t^	3.43 (1.06) *^,i^	3.08 (0.95) *^,i,t^	3.53 (1.08) *^,i^
	**TOT**	3.00 (0.92) *^,i,t^	3.87 (1.01) *^,i,t^	2.69 (0.95) *^,i,t^	3.45 (1.08) *^,i^	3.08 (0.97) *^,i,t^	3.61 (1.10) *^,i^
**Italy**	**M**	3.93 (0.78) *^,f^	3.46 (1.21) *^,†,f^	3.70 (0.90) ^f,t^	3.67 (1.03)	3.53 (1.03) ^f,t^	3.78 (1.03)
	**F**	3.86 (0.82) ^f,t^	3.78 (1.10) ^†,t^	3.61 (0.96) ^f,t^	3.82 (0.99) ^f,t^	3.65 (1.01) *^,f,t^	3.93 (1.05) *^,f,t^
	**TOT**	3.88 (0.80) *^,f,t^	3.68 (1.14) *^,f^	3.64 (0.94) ^f,t^	3.77 (1.00) ^f,t^	3.61 (1.02) *^,f,t^	3.88 (1.04) *^,f,t^
**Turkey**	**M**	4.32 (0.68) *^,f^	3.68 (0.83) *	4.29 (0.67) *^,f,i^	3.69 (0.85) *	4.21 (0.62) *^,f,i^	3.55 (0.92) *
	**F**	4.26 (0.83) *^,f,i^	3.42 (1.04) *^,f,i^	4.25 (0.82) *^,f,i^	3.41 (1.02) *^,i^	4.04 (0.82) *^,f,i^	3.33 (1.07) *^,i^
	**TOT**	4.28 (0.77) *^,f,i^	3.53 (0.97) *^,f^	4.27 (0.76) *^,f,i^	3.52 (0.96) *^,i^	4.11 (0.75) *^,f,i^	3.42 (1.02) *^,i^

Note. Data are reported as mean (standard deviation). M: male participants; F: female participants; TOT: total; * = significantly different by time in the same country; ^†^ = significantly different by gender in the same country; ^f^ = significantly different from France; ^i^ = significantly different from Italy; ^t^ = significantly different from Turkey.

**Table 3:** Descriptive statistics and Bonferroni Post-hoc pairwise comparisons of the interaction time × country in pedagogical formats supporting PE teachers’ behaviors promoting students’ out-of-school PA.

Country	Gender	Text Documents		Slideshows		Videos		Live Streaming Lessons	
		Before	During	Before	During	Before	During	Before	During
France	M	1.98 (1.08)	3.48 (1.27)	1.86 (1.02)	2.76 (1.34)	1.49 (0.86)	3.34 (1.32)	1.11 (0.52)	1.35 (0.86)
	F	2.08 (1.01)	3.32 (1.34)	1.91 (0.92)	2.82 (1.38)	1.65 (0.90)	3.43 (1.25)	1.13 (0.51)	1.30 (0.79)
	TOT	2.03 (1.05) *^,i,t^	3.4 (1.31) *^,t^	1.89 (0.97) *^,i,t^	2.79 (1.36) *^,i^	1.57 (0.88) *^,i,t^	3.38 (1.28) *^,i^	1.12 (0.51) *^,i,t^	1.32 (0.83) *^,i,t^
Italy	M	3.11 (0.99)	3.61 (1.00)	2.94 (1.08)	3.14 (1.15)	2.56 (1.21)	3.70 (1.20)	1.63 (1.08)	2.33 (1.33)
	F	3.08 (1.02)	3.49 (1.13)	2.99 (1.13)	3.00 (1.28)	2.43 (1.19)	3.86 (1.15)	1.66 (1.08)	2.73 (1.58)
	TOT	3.09 (1.01) *^,f^	3.53 (1.09) *^,t^	2.97 (1.11) ^f,t^	3.05 (1.24) ^f^	2.47 (1.2) *^,f,t^	3.8 (1.16) *^,f,t^	1.65 (1.08) *^,f,t^	2.6 (1.51) *^,f^
Turkey	M	3.33 (1.00)	3.01 (1.10)	3.61 (0.91)	3.11 (1.05)	3.55 (0.99)	3.44 (1.07)	2.44 (1.24)	2.79 (1.37)
	F	3.18 (1.11)	2.65 (1.24)	3.40 (1.00)	2.86 (1.16)	3.49 (1.05)	3.21 (1.17)	2.34 (1.23)	2.71 (1.32)
	TOT	3.24 (1.07) *^,f^	2.8 (1.2) *^,f,i^	3.48 (0.97) *^,f,i^	2.96 (1.12) *	3.52 (1.03) ^f,i^	3.3 (1.13) ^i^	2.38 (1.23) *^,f,i^	2.74 (1.34) *^,f^

Note. Data are reported as mean (standard deviation). M: male participants; F: female participants; TOT: total; * = significantly different by time in the same country; ^f^ = significantly different from France; ^i^ = significantly different from Italy; ^t^ = significantly different from Turkey.

**Table 4:** Descriptive statistic and Bonferroni post-hoc pairwise comparisons of the interaction time × country in feedback frequency and its different formats.

Country	Gender	Feedback Frequency		Text Documents		SmartApps		Videos/Pictures	
		Before	During	Before	During	Before	During	Before	During
France	M	2.24 (2.00)	3.32 (1.31)	1.47 (0.90)	2.99 (1.45)	1.17 (0.58)	2.04 (1.36)	1.17 (0.52)	2.10 (1.26)
	F	2.24 (0.95)	3.10 (1.24)	1.53 (0.87)	2.60 (1.50)	1.26 (0.69)	1.98 (1.31)	1.31 (0.68)	2.00 (1.20)
	TOT	2.24 (1.03) *^,i,t^	3.21 (1.28) *^,i^	1.50 (0.88) *^,i,t^	2.80 (1.48) *^,t^	1.21 (0.63) *^,i,t^	2.01 (1.33) *^,i,t^	1.24 (0.6) *^,i,t^	2.05 (1.23) *^,i,t^
Italy	M	2.91 (2.00)	3.65 (0.97)	2.16 (1.12)	2.82 (1.27)	1.81 (0.99)	2.31 (1.29)	1.90 (1.10)	2.77 (1.38)
	F	3.17 (1.10)	4.03 (0.93)	2.15 (1.12)	2.85 (1.24)	1.81 (1.07)	2.34 (1.31)	1.93 (1.11)	3.07 (1.31)
	TOT	3.08 (1.08) *^,f,t^	3.91 (0.96) *^,f,t^	2.15 (1.12) *^,f,t^	2.84 (1.24) *^,t^	1.81 (1.04) *^,f,t^	2.33 (1.30) *^,f^	1.92 (1.10) *^,f,t^	2.98 (1.34) *^,f^
Turkey	M	3.61 (0.83)	3.25 (1.00)	2.92 (1.05)	2.62 (1.20)	2.65 (1.18)	2.62 (1.17)	3.18 (1.12)	2.86 (1.07)
	F	3.48 (0.93)	2.99 (1.17)	2.71 (1.01)	2.34 (1.20)	2.66 (1.12)	2.49 (1.18)	2.83 (1.08)	2.68 (1.21)
	TOT	3.53 (0.89) *^,f,i^	3.09 (1.11) *^,i^	2.79 (1.02) *^,f,i^	2.45 (1.2) *^,f,i^	2.66 (1.14) ^f,i^	2.54 (1.17) ^f^	2.97 (1.10) ^f,i^	2.75 (1.15) ^f^

Note. Data are reported as mean (SD). M: male participants; F: female participants; TOT: total; * = significantly different by time in the same country; ^f^ = significantly different from France; ^i^ = significantly different from Italy; ^t^ = significantly different from Turkey.

## Supplementary Materials

The following are available online at https://www.mdpi.com/1660-4601/17/24/9431/s1, Appendix S1: The questionnaire used for this research; Table S1: RM-MANCOVA 2 Time (before vs. during the lockdown) × 3 Country (France vs. Italy vs. Turkey) × 2 Gender (male vs. female) results for Teachers’ behaviors aiming at promoting students’ out-of-school PA; Table S2: RM-MANCOVA 2 Time (before vs. during the lockdown) × 3 Country (France vs. Italy vs. Turkey) × 2 Gender (male vs. female) results for Pedagogical formats of behaviors aiming at promoting students’ out-of-school PA; Table S3: RM-MANCOVA 2 Time (before vs. during the lockdown) × 3 Country (France vs. Italy vs. Turkey) × 2 Gender (male vs. female) results for frequency and formats of asked feedback about students’ out-of-school PA.
