# Supplementary material for: Promoting Physical Activity during School Closures Imposed by the First Wave of the COVID-19 Pandemic: Physical Education Teachers’ Behaviors in France, Italy and Turkey
Source: Int J Environ Res Public Health. 2020 Dec 16;17(24):9431. doi: 10.3390/ijerph17249431 (PMC7767079; doi:10.3390/ijerph17249431)
Supplement: Supplementary file 1 [file ijerph-17-09431-s001.pdf]

Appendix S1. The questionnaire used for this research.

PE Teachers' Questionnaire

INTRODUCTION:

The COVID-19 pandemic has considerably disrupted our daily routines. In line with containment measures, schools had to be closed in most countries and teachers had to adapt their teaching behaviors. Specifically, Physical Education (PE) teachers, for whom the physical presence of students is especially crucial, are facing the necessity to adapt their practice.

In this context, the present study **investigates PE teachers' behaviors during the lockdown period, mainly in terms of physical activity promotion among your students.** Specifically, this study aims to understand how **teaching behaviors** relative to physical activity promotion, **and their pedagogical formats,** have changed because of the lockdown.

Thereby, the first part of the questionnaire focuses on these behaviors and practices **BEFORE THE LOCKDOWN,** and the second part focuses on these behaviors and practices **DURING THE LOCKDOWN**.

BEHAVIORS TO PROMOTE PHYSICAL ACTIVITY BEFORE THE LOCKDOWN PERIOD

This part of the questionnaire focuses on your behaviors **before** the lockdown period.

1. Before the lockdown, how often did you …

	**Never**	**Rarely**	**Sometimes**	**Often**	**Always**
1. guide your students to engage in out-of-school physical activity?					
2. help them to set personal physical activity goals?					
3. encourage your students to self-monitor physical activity?					

2. Before the lockdown, if you proposed pedagogical contents aiming to promote out-of-school physical activity among your students, in what format and how often did you propose them?

	**Never**	**Rarely**	**Sometimes**	**Often**	**Always**
1. Text documents (e.g. notebooks).					
2. Slideshows (e.g. posters).					
3. Videos (i.e. video tutorials with exercise to do at home).					
4. Live streaming lessons (e.g. online PE classes delivered in live streaming).					

3. Before the lockdown, if you proposed pedagogical contents aiming to promote out-of-school physical activity among your students, how often did you ask them to make some feedback about what they had actually done?

Never	Rarely	Sometimes	Often	Always

4. In what format and how often did you generally ask your students to make these feedbacks?

	Never	Rarely	Sometimes	Often	Always
1. Text documents (e.g. notebooks).					
2. SmartApps (e.g. summary records from smartphone apps).					
3. Pictures and/or videos.					

BEHAVIORS TO PROMOTE PHYSICAL ACTIVITY DURING THE LOCKDOWN PERIOD

This part of the questionnaire focuses on your behaviors **during** the lockdown period.

5. During the lockdown, how often do/did you…

	Never	Rarely	Sometimes	Often	Always
1. guide your students to engage in out-of-school physical activity?					
2. help them to set personal physical activity goals?					
3. encourage your students to self-monitor physical activity?					

6. During the lockdown, if you propose/d pedagogical contents aiming to promote out-of-school physical activity among your students, in what format and how often do/did you proposed them?

	Never	Rarely	Sometimes	Often	Always
1. Text documents (e.g. notebooks).					
2. Slideshows (e.g. posters).					
3. Videos (e.g. video tutorials with exercises to do at home).					
4. Live streaming lessons (e.g. online PE classes delivered in live streaming).					

7. During the lockdown, if you propose/d pedagogical contents aiming to promote outside-school physical activity among your students, how often do/did you ask them to make some feedback about what they have/had actually done?

Never	Rarely	Sometimes	Often	Always

8. In what format and how often do/did you generally ask your students to make these feedbacks?

	Never	Rarely	Sometimes	Often	Always
1. Text documents (e.g. notebooks).					
2. SmartApps (e.g. summary records from smartphone apps).					
3. Pictures and/or videos.					

YOUR PHYSICAL AND MENTAL HEALTH

9. During the last seven days, how would you rate your **physical health**?

Poor	So-so	Good	Excellent

10. During the last seven days, how would you rate your **mental health**, including your mood and your ability to think?

Poor	So-so	Good	Excellent

ADDITIONAL INFORMATIONS

**1.** Are you…	□ **A woman**	□ **A man**
**2.** How old are you?	**……………. years**	
**3.** How long have you been a PE teacher?	**……………. years**	

Table S1. RM-MANCOVA 3 Country (France *vs.* Italy *vs.* Turkey) × 2 Time (before *vs.* during the lockdown) × 2 Gender (male *vs.* female) results for *Teachers' behavior aiming at promoting students' out-of-school PA*

		Wilks' λ	F	df	*p*	η_p^2	Power
Between-subjects	Country	.823	38.809	6, 2270	.000	.093	1.000
	Gender	1.000	.158	3, 1135	.924	.000	.079
	Country × Gender	.982	3.397	6, 2270	.002	.009	.944
Within-subjects	Time	1.000	.164	3, 1135	.921	.000	.080
	Years teach PE × Time	.984	6.200	3, 1135	.000	.016	.964
	Physical health × Time	.998	.845	3, 1135	.469	.002	.235
	Mental health × Time	.997	1.146	3, 1135	.329	.003	.310
	Gender × Time	.999	.271	3, 1135	.847	.001	.102

Note. Years of teaching PE, physical and mental health of participants were entered as covariates.

Table S2. RM-MANCOVA 3 Country (France *vs.* Italy *vs.* Turkey) × 2 Time (before *vs.* during the lockdown) × 2 Gender (male *vs.* female) results for *Pedagogical formats of behaviors aiming at promoting students' out-of-school PA*

		Wilks' λ	F	df	*p*	η_p^2	Power
Between-subjects	Country	.685	59.153	8, 2268	.000	.173	1.000
	Gender	.995	1.556	4, 1134	.184	.005	.484
	Country × Gender	.988	1.678	8, 2268	.099	.006	.742
Within-subjects	Time	.980	5.883	4, 1134	.000	.020	.984
	Years teach PE × Time	.985	4.210	4, 1134	.002	.015	.925
	Physical health × Time	.994	1.657	4, 1134	.158	.006	.513
	Mental health × Time	.994	1.659	4, 1134	.157	.006	.513
	Gender × Time	.995	1.417	4, 1134	.226	.005	.444
	Country × Time × Gender	.989	1.627	8, 2268	.112	.006	.726

Note. Years of teaching PE, physical and mental health of participants were entered as covariates.

Table S3. RM-MANCOVA 3 Country (France *vs.* Italy *vs.* Turkey) × 2 Time (before *vs.* during the lockdown) × 2 Gender (male *vs.* female) results for *Frequency and formats of asked feedback about students' out-of-school PA*

		Wilks' λ	F	df	*p*	η_p^2	Power
Between-subjects	Country	.714	52.050	8, 2268	.000	.155	1.000
	Gender	.993	1.953	4, 1134	.099	.007	.591
	Country × Gender	.974	3.827	8, 2268	.000	.013	.990
Within-subjects	Time	.986	4.116	4, 1134	.003	.014	.919
	Years teach PE × Time	.992	2.246	4, 1134	.062	.008	.660
	Physical health × Time	.994	1.630	4, 1134	.164	.006	.505
	Mental health × Time	.999	.392	4, 1134	.815	.001	.141
	Gender × Time	.995	1.552	4, 1134	.185	.005	.483
	Country × Time × Gender	.988	1.756	8, 2268	.081	.006	.765

Note. Years of teaching PE, physical and mental health of participants were entered as covariates.
